# Medical Department of the Grosvenor Library.—A New Arrangement to Facilitate Research

**Published:** 1904-11

**Authors:** 


### CORRESPONDENCE.

### Medical Department of the Grosvenor Library.—A New Arrangement to Facilitate Research.

*Editor Buffalo Medical Journal:*

SIR.—Cards, like the enclosed, are being sent to the practising physicians of Buffalo in the hope that by their use we may make the medical department of this library more useful to the profession in this city. Our medical department is being constantly increased and strengthened by the addition of the best of the new medical books as they appear, and it is now in a better position than ever before to be of assistance to the student. If you wish to undertake the investigation of any subject here at the Grosvenor Library, or the preparation of any paper, fill out one of the enclosed cards and send it to the library a few days before you need the material, and we will use our best endeavors to have the resources of the library on that subject ready for you. We have found that the use of these cards in other departments of the library saves the time both of the student and of the library attendants, and the results of their use on the whole have been found very satisfactory. We hope the same may prove true of the medical department.

The use of the medical department, like that of all other departments of the library work, is entirely free to the physicians of this city and vicinity, and a cordial invitation is extended to all to avail themselves of its advantages.

The library is open from 9.00 a. m. to 10.00 p. m. during the week, and from 2.00 to 6.00 p. m. on Sundays. During the day there is a special attendant on the balcony floor where the medical department is located.

EDWARD P. VAN DUZEE,
*Librarian.*

BUFFALO, October 15, 1904.

The card referred to is as follows:

GROSVENOR LIBRARY, BUFFALO, N. Y.

Application by..........Address..........For material on the subject named below:..................The applicant will call to consult this material on or about....................

If the applicant will fill in the above blanks, giving name, address and the subject on which material is desired, the library attendants will look up the matter in advance, so the resources of the library on this subject will be at the disposal of the student at the time named above.

